# Structure Based Design and Synthesis of Peptide Inhibitor of Human LOX-12: *In Vitro* and *In Vivo* Analysis of a Novel Therapeutic Agent for Breast Cancer

**DOI:** 10.1371/journal.pone.0032521

**Published:** 2012-02-23

**Authors:** Abhay kumar Singh, Ratnakar Singh, Farhat Naz, Shyam Singh Chauhan, Amit Dinda, Abhay Anand Shukla, Kamaldeep Gill, Vaishali Kapoor, Sharmistha Dey

**Affiliations:** 1 Department of Biophysics, All India Institute of Medical Sciences, New Delhi, India; 2 Department of Biochemistry, All India Institute of Medical Sciences, New Delhi, India; 3 Department of Pathology, All India Institute of Medical Sciences, New Delhi, India; 4 Department of Biotechnology, All India Institute of Medical Sciences, New Delhi, India; Aligarh Muslim University, India

## Abstract

Human breast cancer cell proliferation involves a complex interaction between growth factors, steroid hormones and peptide hormones. The interaction of growth factors, such as epidermal growth factor (EGF), with their receptors on breast cancer cells can lead to the hydrolysis of phospholipids and release of fatty acid such as arachidonic acid, which can be further metabolized by cyclooxygenase (COX) and lipoxygenase (LOX) pathways to produce prostaglandins. The high concentration of prostaglandins has been associated with chronic inflammatory diseases and several types of human cancers. This is due to the over expression COX, LOX and other inflammatory enzymes. Ten peptides were designed and synthesized by solid phase peptide synthesis and analyzed *in vitro* for enzyme inhibition. Out of these peptides, YWCS had shown significant inhibitory effects. The dissociation constant (K_D_) was determined by surface plasmon resonance (SPR) analysis and was found to be 3.39×10^−8^ M and 8.6×10^−8^ M for YWCS and baicalein (positive control), respectively. The kinetic constant Ki was 72.45×10^−7^ M as determined by kinetic assay. The peptide significantly reduced the cell viability of estrogen positive MCF-7 and estrogen negative MDA-MB-231 cell line with the half maximal concentration (IC_50_) of 75 µM and 400 µM, respectively. The peptide also induced 49.8% and 20.8% apoptosis in breast cancer cells MCF-7 and MDA-MB-231, respectively. The YWCS was also found to be least hemolytic at a concentration of 358 µM. *In vivo* studies had shown that the peptide significantly inhibits tumor growth in mice (p<0.017). This peptide can be used as a lead compound and complement for ongoing efforts to develop differentiation therapies for breast cancer.

## Introduction

LOX plays an essential role in the biosynthesis of leukotrienes (LTs). LTs are potent biological mediators in the pathophysiology of inflammatory diseases and host defense reactions. These properties imply a significant role for LTB4 in the pathogenesis of inflammatory diseases such as asthma, atherosclerosis and cancer [1]–[4]. The metabolism of arachidonic acid via COX or LOX pathway generates eicosonoids which has been implicated in the pathogenesis of a variety of human diseases, including cancer, and may play important roles in tumor promotion, progression and metastasis. The involvement of LOX-12 expression and function in tumor growth and metastasis has been reported in both murine and human tumor cell lines [5]. LOX-12, product 12-HETE is one of the most important lipid metabolites to influence tumor progression [6]. It has been reported that LOX-12 is over expressed in tumor tissues including prostrate, breast, colorectal and lung cancer [7]–[10]. The tumor suppressive and anti-angiogenesis effects of LOX-12 inhibitors may provide a new approach to the treatment of human breast cancer. The development of peptides as drugs is increasingly attracting the attention of pharmaceutical companies. The advantages of peptides as drugs include their high specificity, potency, and activity. These peptides may be responsible for molecular recognition and other biological processes. Small peptides as a drug are very specific in nature. Also, peptide drugs pose other advantages over therapeutic proteins, owing to their higher solubility, better stability, more bio-availability and negligible immune response. This study reports, *in vitro* and *in vivo* evaluation of peptide inhibitors against human LOX-12.

## Materials and Methods

### Ethics Statement

The volunteers of the study provided written informed consent and the Ethics Committee of All India Institute of Medical Sciences (AIIMS) approved the study protocol and the permit number is A-9/25.07.2007. The *in vivo* study was carried out in strict accordance with the recommendations in the guide for the care and use of laboratory animals of the AIIMS. The protocol was approved by the committee on the ethics of animal experiments of AIIMS (Permit Number: 549/IAEC/10).

### Cloning, expression, purification and characterization of human LOX-12

The total RNA was isolated from MCF-7 cell line (National centre for cell sciences, Pune, India) and converted to total cDNA by using reverse transcriptase. The specific primer for human LOX-12 was used for amplification and cloned into pGEMT easy cloning vector (Promega) and subcloned in pET28a bacterial expression vector. E. coli BL21 codon+ competant cells (Novagen) were transformed with the expression vector containing His-tagged LOX-12 using standard Novagen procedure. The cells were grown at 310K in Luria-Bertani (LB) medium containing 50 µg/ml Kanamycin to an absorbance of 0.6 at 600 nm. The expression was induced by the addition of 1 mM isopropyl-β-D-thiogalactosidase (IPTG). The cells were grown for 4 h at 37°C and centrifuged at 8,000 g for 10 min. The cell pellet was resuspended in 10 ml of buffer (20 mM Tris-HCl pH–8.0, 150 mM NaCl). The cells were lysed by using sonicator(Sonics) using 5 sec pulse On and 9 sec Off for 5 minute and centrifuged at 12,000 g for 20 min to remove the inclusion bodies. The protein was purified from inclusion bodies under denaturing condition using urea and refolded by passing through the column of Ni-NTA-Agarose (5 ml, QIAGEN) equilibrated in binding buffer. The bound protein was eluted with buffer containing 300 mM imidazole. The protein was characterized by SDS-PAGE using Laemmli system of buffers [11] and was then subjected to western blotting. Gels were electroblotted (Protean Trans blot cells; Bio-Rad) onto nitrocellulose membranes. The human LOX-12 primary antibody (1∶500) and secondary antibody (anti-goat alkaline phosphatase-conjugated; Santa Cruz Biotechnology, Inc) were used for development of the blot.

### Activity assay of Recombinant Human LOX-12

The activity of purified recombinant LOX-12 was determined by using the conjugated diene method of biochemical assay. Enzyme activity was indirectly measured by estimating the rate of product formation. Hydroperoxy lipid product of the reaction contains a conjugated diene which strongly absorbs at 234 nm. The purified enzyme was used for activity assay. The assay mixture contained 20 µM arachidonic acid (substrate), 0.2% Tween-20, 50 mM potassium phosphate buffer pH 7.2 and 1.0 µM purified enzyme. The change in absorbance at 234 nm was observed and the activity was calculated.

### Synthesis of Peptides

The peptides were synthesized by solid phase peptide synthesizer PS3 (Protein technology, USA) using Fmoc and Wang resin chemistry [12]. The solvent used for the synthesis was dimethylformamide (DMF). 2-(1H-Benzotriazole-1-yl)-1,1,3,3-tetramethyluronium hexafluorophosphate (HBTU) was used as an activator of the Fmoc amino acids (Chem Impex, USA). Fmoc was deprotected by 20% piperidine and wang resin was cleaved by Trifluoroacetic acid (TFA). The peptides were precipitated from dry ether.

### Analytical RP-HPLC of Peptides

The purity of peptides was verified by analytical RP-HPLC, C18 reversed phase column (RPC) (1.6×10 cm, Amersham Bioscience). 1 mg/mL of peptide was loaded to the RPC. The linear gradients were formed by passing two different solvents, where solvent A was 0.05% aqueous TFA, pH 2 and solvent B was 0.05% TFA in acetonitrile. The flow rate was 0.25 mL/min at room temperature. The molecular weights were confirmed by MALDI-TOF.

### Kinetics and inhibition studies of the peptides by spectrophotometer

For inhibition study, the human LOX-12 was incubated with peptide for 30 min, which was then added to the reaction buffer containing arachidonic acid (substrate), 0.2% Tween-20, 50 mM potassium phosphate buffer pH 7.2. The change in absorbance was monitored at 269 nm and the percentage of inhibition was calculated. A control reaction was always carried out without any peptide inhibitor to ensure the activity of protein under experimental conditions.

For kinetic assay, the above experiment was done with four different concentrations of peptides (25–100 µM). For each concentration of peptide the experiment was repeated with six different concentrations (20–120 µM) of substrate, arachidonic acid. The enzyme concentration was constant (1.0 µM) throughout the assay. The competitive kinetic constant (Ki) was calculated graphically using the Michaelis-Menton equation in graphpad prism software. The graph of activity vs. concentration of arachidonic acid was plotted.

### Binding studies of peptides with purified LOX-12 by SPR

The binding interaction between LOX-12 with peptides was performed using a biosensor based on SPR. The interaction phenomenon of two biological molecules can be monitored directly by the SPR. The phenomenon of SPR was studied by Otto [13] and Kretschmann and Raether [14] and it was used as a chemical detection method by Nylander et al [15]. An automatic instrument BIAcore 2000 (Pharmacia Bioscience) was used. Six histidine-tag which were attached to the N-terminal position of LOX-12 was an ideal tag for immobilization due to strong rebinding effect caused by the high surface density of immobilized Ni^2+^–nitriloacetic acid (NTA) on the chips. The binding of analyte i.e., the peptide in solution can be studied by monitoring the change in the resonance unit (RU) values of the sensorgram, where the progress of the interaction was plotted against time, revealing the binding characteristics. Analysis of binding property i.e., the association constant (K_A_) for the formation of multi-molecular complex and dissociation constant (K_D_) were achieved in very short time and with a small amount of samples. First the flow cell was activated by passing nickel chloride and 60 µl of His-LOX-12 (50 µg/ml) was injected over the flow cell at the flow rate of 5 µl/min. 350 RU of LOX-12 was immobilized under these conditions, where 1 RU corresponds to immobilized protein concentration of ∼1 pg/mm^2^. The analyte i.e., peptide inhibitors at a concentration of 25 µM were passed over the immobilized LOX-12 at a flow rate of 10 µl/min and the sensogram was run for 4 min, likewise two more concentrations i.e, 50 and 75 µM of peptides of same volume were passed over the chip and the change in sensogram was observed. The graph shows the change in RU values with time for different concentration of peptides. The rate constants K_A_ and K_D_ were obtained by fitting the primary sensorgram data using the BIA evaluation 3.0 software. The dissociation rate constant is derived using equation:
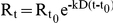
Where, R_t_ is the response at time t, R_to_ is the amplitudes of the initial response and K_D_ is the dissociation rate constant. The association rate constant, K_A_ can be derived using equation given below from the measured K_D_ values.
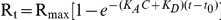
Where, Rt is the response at time t, Rmax is the maximum response, C is the concentration of the analyte in the solution.

### Measurement of Minimal hemolytic concentration (MHC)

The effect of peptide on hemolysis was determined by using human red blood cells (hRBC from volunteer). The freshly collected hRBC were centrifuged for 10 mins to remove the buffy coat and washed with phosphate buffered saline (PBS: 35 mM Na_2_HPO_4_, pH 7.0 and 150 mM NaCl). 100 µl of the hRBC (suspended in 1% (v/v) in PBS) and 10 µl peptide solution were added into the sterile 96 well plates in triplicate. The plates were incubated for 1 h at 37°C and centrifuged at 1000× g for 5 mins. The supernatant were transferred to fresh 96 well plates, where hemoglobin released was monitored by measuring the absorbance spectrophotometrically at 541 nm. Similar steps were carried out for Gentamycin, as a control. The percentage of hemolysis (determined in PBS and 0.1% Triton X-100) was calculated from the standard graph which was plotted with % hemolysis of prepared RBCs vs optical densities.

### Tumor cell cytotoxicity of the inhibitory peptides by MTT assay

Cytotoxic effect of LOX-12 inhibitory peptides was analyzed on MCF-7 (ER+) and MDA-MB-231 (ER+) breast cancer cell lines by MTT [3-(4, 5-dimethylthiazol-2-yl)-2, 5-diphenyltetrazolium bromide] dye reduction assay. Briefly, 3×10^3^ cells/100 µl media were seeded in 96-well plates 24 h before the experiment. The cells were then incubated with different concentrations of the peptides for 24, 48 and 72 h as indicated. The cells were treated with DMSO (same concentration as used to solubilise the peptide) which was then subtracted from all the cytotoxic values.10 µl/well MTT-solution (10 mg/ml in PBS) was then added and plates were further incubated for 3 h at 37°C. The formazan crystals formed were dissolved by adding 100 µl/well of dimethyl sulfoxide (DMSO). Absorbance was measured by a microplate reader at 570 nm with a reference filter 650 nm. 100 µl of medium with 10 µl of MTT stock-solution and 100 µl of DMSO was used as a blank solution to correct the background. The data obtained was presented as percentage viability in the best-fit (linear) dose response curve. The IC_50_ values at 95% confidence intervals was calculated for all cell lines. Each concentration was used in triplicate. The peptide AIRS was used as a negative control. The MTT assay of peptide AIRS is submitted as a supplementary data [Figure S1].

### Assessment of apoptosis by annexin-V binding assay and Flow Cytometry

The frequency of apoptosis in MCF7 cells and MDA-MB-231 by the treatment with YWCS was assayed by annexin-V binding assay. Briefly, MCF7 cells and MDA-MB-231 were pulsed with the peptide for 72 h. After termination of the culture, cells were stained with annexin-V apoptosis detection kit (Biolegend) according to the manufacturer's instructions. The cells were washed twice with PBS, and resuspended in annexin-V binding buffer. To the cell suspension, 5 µl of FITC-conjugated annexin-V (Biolegend) and 10 µl of propidium iodide (PI) (50 µg/ml) solution were added and further incubated for 15 min at room temperature. About 10,000 events were acquired in FACS Canto flow cytometer (Becton–Dickinson, USA). The frequency of annexin-positive (apoptotic) cells was determined using BD FACSDiva software.

### FITC labeling of peptides and their intracellular localization

For labeling of peptides with Flourescein isothiocyanate (FITC), 1∶1 equivalent of NH_2_-YWCS-wang resin and FITC in DMF were stirred together overnight. The peptide labeled with FITC was then cleaved from the resin using the standard procedure. To evaluate the localization of the inhibitory peptide in MCF-7 and MDA-MB231 cell line, cells were seeded on cover slips in 6-well plates and allowed to adhere for 24 h. They were then treated with the FITC-labeled peptide and incubated further for 48 h. The cells were then washed with PBS, fixed with 4% paraformaldehyde for 15 min and stained with 50 µg/ml PI for nuclear staining. The cells were visualized under Confocal microscope (Nikon) for green (FITC) and red (PI) fluorescence to check the intracellular localization of the peptides.

### Preliminary study of peptide action in tumor mice model

*In-vivo* animal experimentation of peptide for anticancer activity was done using mice model. Swiss albino mice were obtained from Central animal facility, AIIMS at 55–65 days of age. The animals were housed in polycarbonated cages, bedded with husk. The animal facility was environmentally controlled; mice were maintained at normal room temperature with suitable relative humidity and 12 h light, 12 h dark cycle. Animals were fed chow food and purified water ad libitum throughout the study. Animals were randomized into 5mice per cage in 3 groups (Group I, II and III) for the study.

### Tumor Induction

Ehrlich's Ascites Tumor cell line (EAT) (murine breast carcinoma), was used for tumor induction. The cell line was maintained *in-vivo* by passaging weekly intraperitonealy (i.p) from mice to mice. For tumor induction, 14 million cells approximately 150 µl volumes was injected to the animal in a group subcutaneously on the dorsal side of the body (back) and kept under observation for tumor onset.

Tumor size was recorded by vernier calipers and tumor volume was calculated using the formula: V = 0.5×L×W^2^, where L is the length (long axis) and W is the width (short axis).

### Evaluation of peptide action after injection in experimental mice

In Group I (control, untreated) only tumor cell line was administered. In group II, YWCS peptide was administered when the tumor volume reached 0.22±0.10 cm^3^ on day 10, at a dose of 200 µmol intravenously (i.v) per mouse once daily for 20 days from day 11 to day 30. In group III, same concentration of peptide was injected intravenously (i.v) simultaneously after injecting tumor cells subcutaneously on day one. During experimental period mice were under observation till sacrificed.

### Statistical analysis

The statistical analysis was carried out using the Graphpad Instat 3 software and p<0.05 was considered statistically significant. For the comparison of the findings, paired and unpaired t-test was performed.

## Results

### Purification and Characterization of Human LOX-12

**Figure 1:**
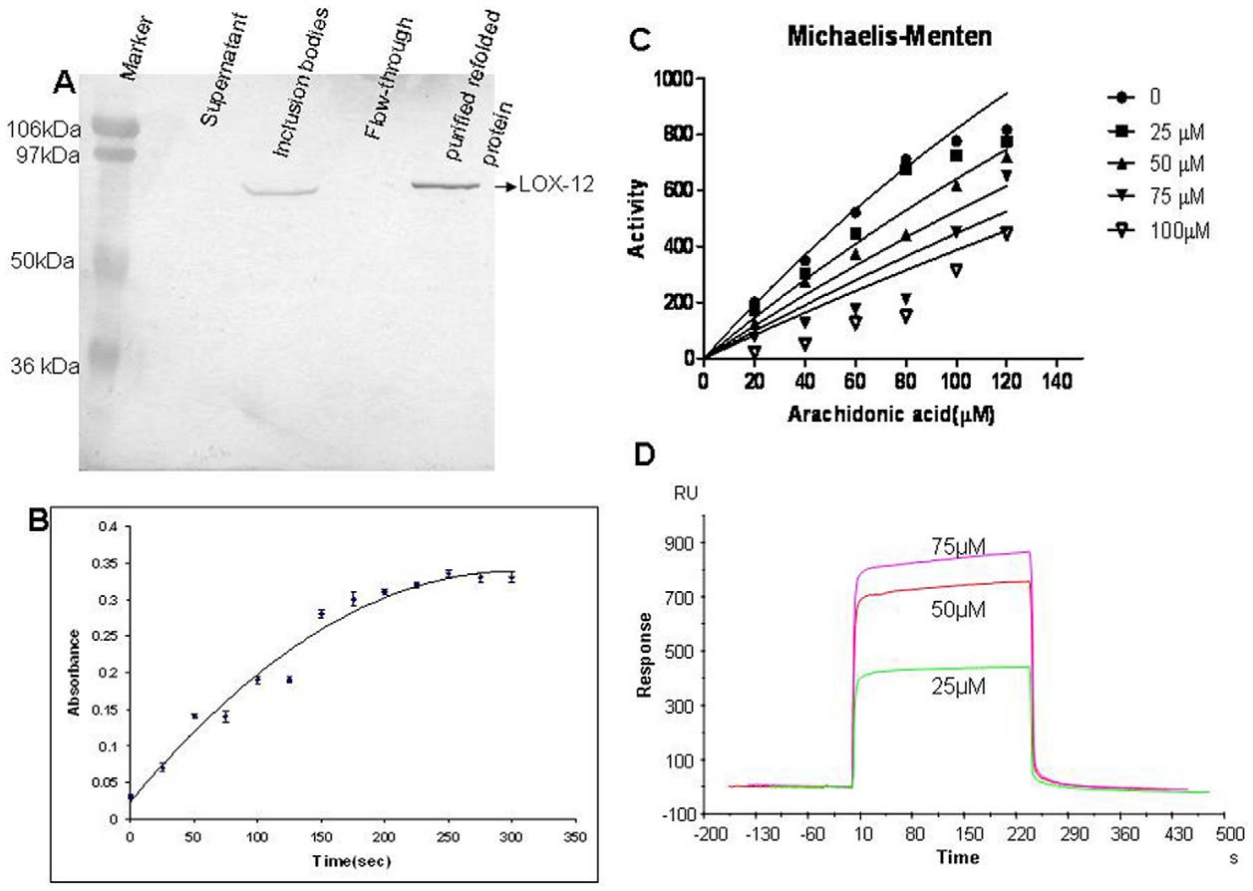
Western blot showing LOX-12 expression: (**A**) **Molecular weight marker, supernatant of bacterial extract, inclusion bodies of bacterial cell extract, flow-through of the Ni-NTA column, purified refolded protein,** (**B**) **Activity profile of purified LOX-12.** (**C**) Michaelis-Menten plot of LOX-12 activity versus arachidonic acid concentration showing decrease in activity at different concentrations (0, 25, 50, 75,100 µM) of YWCS. (**D**) Sensogram showing binding of different concentrations of YWCS (25, 50 and 75 µM) on immobilized with His-LOX-12 over the Ni^2+^-NTA chip.

Human LOX-12 protein was purified from inclusion bodies by the method of on-column refolding as described in the material and methods section. Approximately 20 mg of protein was recovered from the Ni^2+^-NTA affinity column. In SDS-PAGE approximately 90–95% purity of the protein was observed (data not shown). This was followed by western blotting showing the presence of LOX-12 in the inclusion bodies of the bacterial cell extract (**Figure 1A**).

### Activity Assay of Human LOX-12

The activity of purified recombinant LOX-12 was determined by using the conjugated diene method of biochemical assay. Enzyme activity was indirectly measured by estimating the rate of product formation. The graph (activity profile) of absorbance vs. time is shown in F**igure 1B**.

### Screening of peptides

The activity assay for LOX-12 in presence of 10 different peptides with substrate was performed by using spectrophotometer. The assay was performed at 269 nm which gives the absorption maxima for the product LTB4. Table 1 shows the percentage inhibition for the 10 peptides. The peptide YWCS showed maximum inhibition of 80%.

**Table 1:** The peptides screened as inhibitors of LOX-12.

Serial number	Peptide Studied	% inhibition
1	YWG	71.6
2	AIYW	69.3
3	YW	73.2
4	FWY	80.4
5	FYS	79.8
6	YWCS	86.1
7	WFA	73.5
8	WFC	72.1
9	WKS	53.2
10	FWCS	74.2
11	Baicalein	85.1

Inhibition assays were performed in triplicates and average values have been reported.

YWCS was found to be inhibit the activity of protein by 86.1%.

### Determination of kinetic constant (Ki)

Competitive kinetic constant of the best peptide YWCS was calculated using graphpad prism software. A linearised analysis of the data (Michaelis-Menten equation) [16] showed a competitive mode of inhibition (**Figure 1C**). The Ki value obtained for the peptide YWCS was 72.45×10^−7^ M. The direct plots of reaction velocity versus substrate concentration demonstrated classical steady-state kinetic behavior.

### SPR analysis

The plot (**Figure 1D**) shows the sensorgram for the binding of the varying concentrations of the peptide. The changes in RU with varying concentration of peptide showed the change of mass on the LOX-12 immobilized on chip with time. The binding of peptide YWCS with LOX-12 was the strongest due to the faster on (association), K_A_ = 2.95×10^7^ M was well as slower off rate (dissociation) K_D_ = 3.39×10^−8^ M.

### Assay for hemagglutinating activity

**Figure 2:**
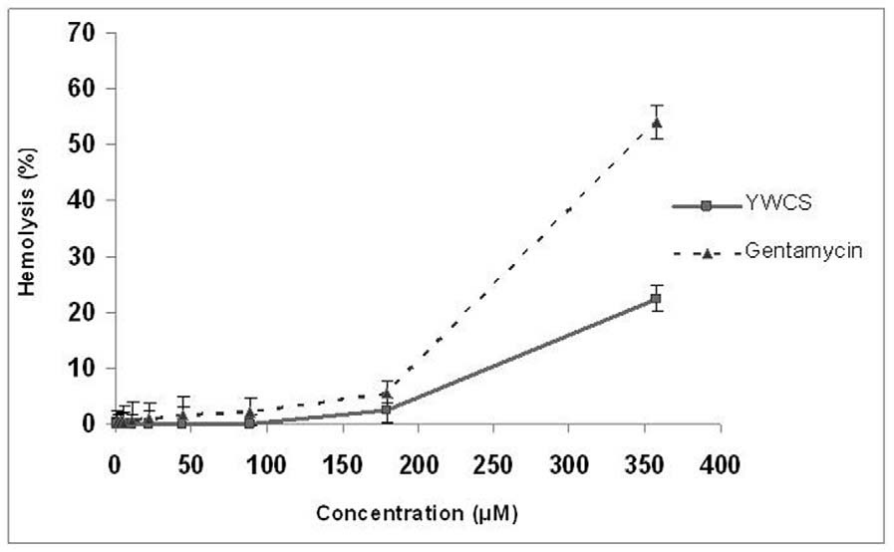
Graph showing percentage of hemolysis with different concentrations (0–358 µM) of YWCS and of gentamycin as a control.

The haemolytic activity of the peptide was determined to ensure the toxicity to erythrocytes using human RBCs. The concentration upto 358 µM of the peptide lysed only 22% human erythrocytes (**Figure 2**).

### Cytotoxic activity of peptides on breast cancer cell line

**Figure 3:**
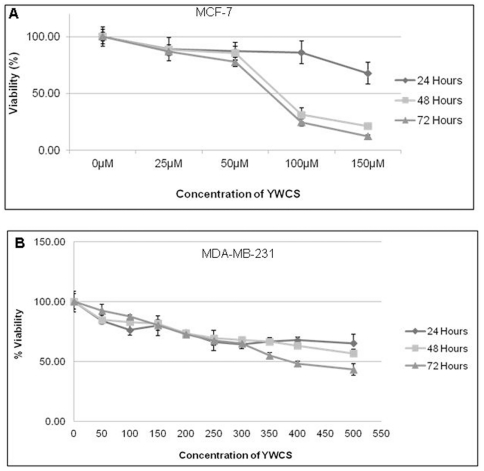
Dose-response curve: (**A**) **showing % viability of MCF-7 cells at 0–150 µM concentrations of YWCS (0.05% DMSO as a vehicle control), for three different time points (24, 48 and 72 h). IC_50_ was found to be 75 µM at 72 h.** (**B**) showing % viability of MDA-MB-231 cells at 0–500 µM concentration of YWCS (0.05% DMSO as a vehicle control), for three different time points (24,48 and 72 h). IC_50_ was found to be 400 µM at 72 h.

MCF-7 cells were treated with 0–150 µM (0.05% DMSO as a vehicle control) and MDA-MB-231 was treated with 0–500 µM (0.05% DMSO as a vehicle control) of the LOX-12 inhibitory peptides for 24, 48 and 72 h to perform the cytotoxic activity. The half maximal concentration (IC_50_) of 75 µM was obtained which represents the concentration of the peptide at which the cell growth is inhibited by 50%. The percentage cells viability by peptide at concentrations 25, 50, 100, 150 µM was 88.6, 85.7, 31.2, 21.1% respectively at 72 hrs. In case of MDA- MB 231, the IC_50_ value was 400 µM. The percentage of cell viability after the treatment of peptide at concentrations 50, 100, 150,200,250,300,350,400 and 500 µM was 92, 87.46, 80.36, 72.51, 7.37,64.95, 54.76, 48.19, 43.07% respectively at 72 h. The dose-response curves have been shown in **Figure 3a and 3b**.

### Apoptotic activity of YWCS on MCF-7 and MDA-MB-231 cells

**Figure 4:**
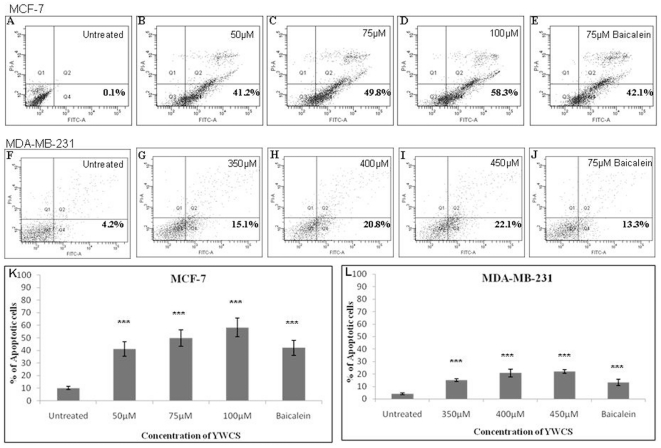
Apoptotic analysis of cancer cells with YWCS by flow cytometry; MCF-7 cells: (**A**) **untreated cells (0.05% DMSO as a vehicle control)**, (**B–D**) **treated with 50, 75 and 100 µM YWCS respectively**, (**E**) **treated with baicalein.** MDA-MB231 cells :(**F**) untreated cells (0.05% DMSO as a vehicle control), (**G–I**) treated with 350,400 and 450 µM YWCS respectively,(**J**) treated with baicalein. (**K**) and (**L**) bar diagram showing mean percentage of apoptotic cells of the above cell lines treated and untreated with peptide and baicalein.[*** represent the comparison of bar of treated cells with the untreated cells].

The apoptotic activity of the peptide YWCS was performed using annexin-V binding assay. Apoptosis induction was observed as early as 72 h. In this experiment MCF-7 cells were treated with 50, 75 and 100 µM peptide YWCS (0.05% DMSO as a vehicle control) and the mean percentage apoptotic cell were 41.2±5.8, 49.8±6.5 and 58.3±7.5, respectively, The mean percentage of apoptotic cell were 15.0±1.2, 20.8±3.2 and 22.1±1.5 treated with 350, 400 and 450 µM of peptide concentrations (0.05% DMSO as a vehicle control) respectively in MDA-MB-231 cells, The frequency of apoptotic cells was significantly higher in peptide-treated cells; MCF-7 (p<0.0001) and MDA-MB231 (p<0.0010) compared to the untreated controls The frequency of the cells in the early phase of apoptosis for all the treatments is shown in **Figure 4**.

### Intracellular localization of FITC labeled peptides

**Figure 5:**
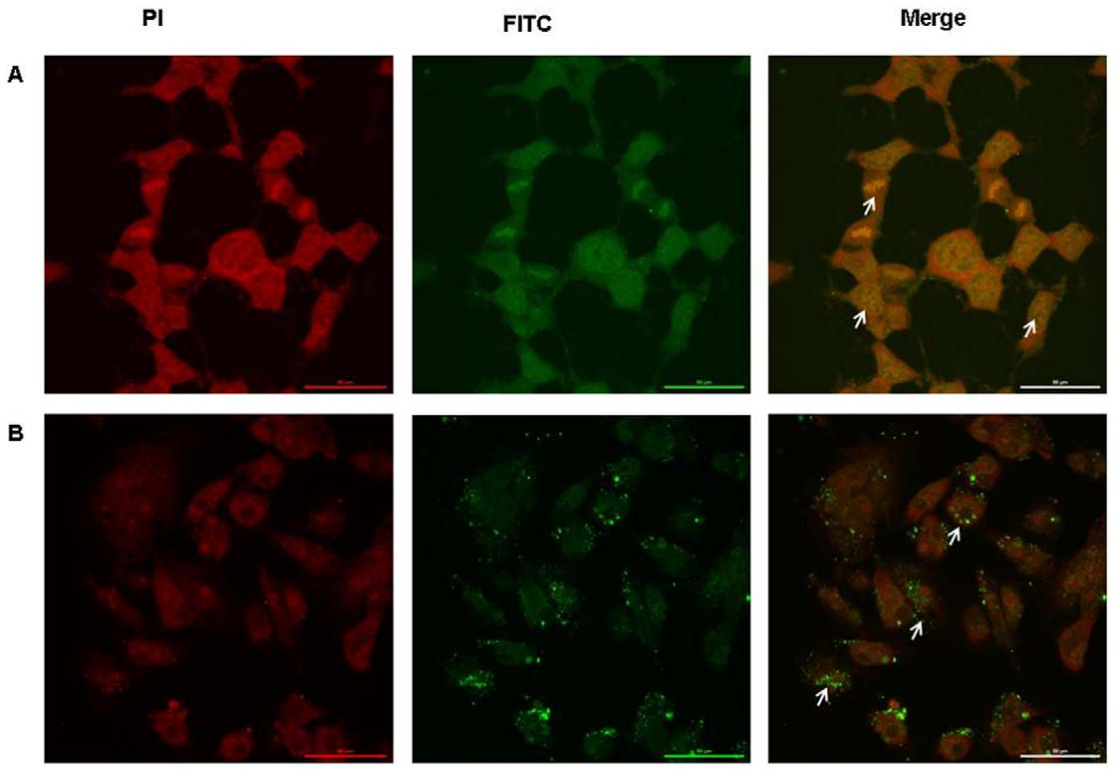
Confocal microscopy pictures showing localization of FITC-labelled YWCS in MCF-7 and MDA-MB-231 cells. White arrows in the merged image show that the peptide enters the cell cytoplasm. (**A**) MCF-7 cells treated with 75 µM YWCS. (**B**) MDA-MB-231 cells treated with 400 µM YWCS peptide.

YWCS was tagged with FITC to observe its intracellular localization in MCF-7 and MDA-MB-231 breast cancer cell lines. The fluorescein-conjugated peptide was seems to be entered to cytoplasmic regions of the cells. (**Figure 5**).

### *In vivo* study: Efficacy of YWCS peptide for antitumoral activity administrated on Swiss albino mice implanted with EAT tumor cell line

**Figure 6:**
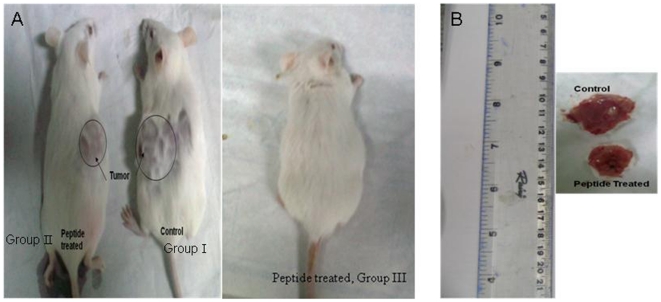
In vivo experiments showing effect of YWCS on tumor initiation and progression: **(A)- Group I; untreated mice, Group II; treated mice with peptide after 20 days of treatment (treatment started after 10 days of tumor growth), Group III; peptide induce simultaneously with EAT tumor cells showing no tumor initiation and (B)The tumor size of Group I and Group II mice after sacrifice.**

During the period of study, there was no significant variation in the weight of animals. The antitumor effect of YWCS peptide administration on Swiss albino mice implanted with EAT tumor cell line are shown in Table 2. All the control (untreated mice) showed progressive increase in tumor volume, reaching 2.09±0.06 cm^3^ on day 20 to 4.5±0.24 cm^3^ on day 30 (Fig. 6a). In contrast, the administration of YWCS peptide 200 µmol intravenously (i.v) once daily for 20 days from day 11 to day 30 in group II, the tumor volume reached 1.62±0.49 cm^3^ on day 20 and 3.04±1.06 cm^3^ on day 30 (Fig. 6a). A significant (p<0.017) difference was observed in the tumor volume between group I and group II mice on day 20 and day 30 (Fig. 6a).

**Table 2:** Antitumor activity on YWCS peptide on EAT tumor bearing mice.

Groups and treatment		Tumor volume, cm^3^		
		Day 10	Day 20	Day 30
Group I	Control* (non treated)	0.048±0.0009	2.09±0.06	4.5±0.24
Group II	YWCS peptide treated 200 µmol from day 11–30	0.22±0.10	1.62±0.49	3.04±1.06
Group III	EAT tumor cell line+peptide injected (i.v)	No tumor onset	No tumor onset	No tumor

200 µmol of YWCS peptide was intravenously (i.v) administered simultaneously after injecting EAT cell line at day one in group III animals and kept them under observation for 20 days. No tumor was observed in this group (Fig. 6b). A significant variation was observed between group I and group III on day 20. Group I (control) showed tumor initiation and progression both, in contrast, group III (peptide treated) showed no tumor initiation.

In addition to this, Group I (control) and group II (peptide treated for 20 days) showed liver and spleen enlargement in all the sacrificed animals, but in group III, the size of liver and spleen was similar to the healthy mice. Preliminary study of YWCS peptide action showed that it could significantly suppress the growth of tumor as well as prevented the initiation of tumor.

## Discussion

There are three isoform of human LOX i.e; LOX-5, LOX-12 and LOX-15. It has been reported earlier that LOX-12 is over expressed in breast cancer [9]. The structure of active site of 5, 12 and 15 LOX are similar. The inhibitor of LOX-5 like curcumin and zileuton are anti-cancer agent [17]–[18]. Baicalein, which is the specific inhibitor of LOX-12 showed anti cancer activity in different cancer cell line [19]. Though the complex structure of LOX-12 with inhibitor is not known, the available complex structure with other LOX had shown that the presence of an aromatic moiety with hydroxyl group and a hydrophobic residue in the inhibitor are important for the affinity towards the active site residue of LOX [20]–[21]. Earlier we have reported FWY as a strong inhibitor of soybean LOX [22] In this study ten peptides were screened for inhibition (Table 1).The first five peptides in table 1 were reported in our previous paper [22] which had already shown a good percentage of inhibition with soybean LOX-3. So we carried out their inhibition test with human LOX-12. The other five peptides were also designed keeping the two key features in mind i.e; presence of an aromatic moiety with hydroxyl group and hydrophobic residues which are important moieties for the affinity to the active site of the protein.

The peptide YWCS showed highest affinity to LOX-12 among the other peptides (Table 1). This peptide was confirmed to be a competitive inhibitor in the presence of substrate. The binding affinity of this peptide with LOX-12 was also confirmed by SPR technology, where the dissociation constant was approximately in nanomolar concentration range which was much higher than the known inhibitors of LOX-12.

The LOX-12 metabolite, 12-HETE, has been reported to increase the proliferation and invasion of breast cancer cells, by mechanisms such as induction of collagenase secretion from the cell [23]. This effects of LOX-12 can be blocked by inhibitors of LOX-12 [24]–[25].

The association of breast cancer and estrogen receptor is very well known. The breast cancer patients with ER+ have been reported to have positive response to chemotherapy and good prognosis [26]–[28]. Accordingly both the breast cancer cell line MCF-7 (ER+) and MDA-MB-231 (ER−) were used to see the effect of peptide on it. The peptide potentially inhibited the cell growth of MCF-7 (ER+) and MDA-MB-231(ER−) in a dose and time –dependent manner and also induced apoptosis in both the breast cancer cell lines. Hence, cell death observed in breast cancer cells treated with peptide inhibitor was via apoptosis induction, which appear to be independent of the hormone receptor status. The invasive breast cancer cell line MDA-MB231 expressed higher level of LOX-12 as compared to MCF7 [29]. The dose of peptide required for the cytotoxicity and apoptosis was higher in case of MDA-MB231 compare to MCF-7. This difference in dosage of peptide inhibitor of LOX-12 may be assumed due to the difference in expression of LOX-12 in MDA-MB231 and MCF-7 cell line. The exact reason yet to be elucidated.

The appearance of permeabilized cells labeled with fluorescein-conjugated peptide suggests that the peptide was internalized and resided in the cytoplasmic region of the MCF-7 and MDA-MB231cells. The peptide was proved to be specific inhibitor of cystosolic enzyme LOX-12.

It was found that the peptide YWCS prevented the initiation of tumor growth in mice when it was injected simultaneously with tumor cell and it also slow down the tumor progression significantly when the peptide dose was administered after 10 days of tumor growth. Thus we can speculate that LOX-12 under *in vivo* conditions is more important for the progression of the disease.

It can be summarized that the tetrapeptide designed on the basis of the structure of LOX-12 is a potent inhibitor of LOX-12 and can be a selective anti breast cancer agent due to its strong anti-cancer property. The hemolytic studies showed that the peptide was nearly non-toxic to human erythrocytes. This aspect of the peptide showed that it can be delivered via the intravenous route, although further evaluation is necessary.

In light of the above discussion we may state that YWCS can surely set a platform for the development of a promising anti cancer drug in future. This peptide can be use as a lead compound and complement for ongoing efforts to develop differentiation therapies for breast cancer.

## Supporting Information

Figure S1 MTT assay of peptide AIRS (Negative control).
(DOC)

## References

[1] Nakano H, Inoue T, Kawasaki N, Miyataka H, Matsumoto H (2000). Synthesis and biological activities of novel antiallergic agents with 5-lipoxygenase inhibiting action.. Bioorg Med Chem.

[2] Harats D, Shaish A, George J, Mulkins M, Kurihara H (2000). Overexpression of 15-lipoxygenase in vascular endothelium accelerates early atherosclerosis in LDL receptor-deficient mice.. Arterioscler Thromb Vasc Biol.

[3] Steele VE, Holmes CA, Hawk ET, Kopelovich L, Lubet RA (1999). Lipoxygenase inhibitors as potential cancer chemopreventives.. Cancer Epidemiol Biomarkers Prev.

[4] Avis I, Hong SH, Martinez A, Moody T, Choi YH (2001). Five-lipoxygenase inhibitors can mediate apoptosis in human breast cancer cell lines through complex eicosanoid interactions.. Faseb J.

[5] Ikemoto S, Sugimura K, Kuratukuri K, Nakatani T (2004). Antitumor effects of lipoxygenase inhibitors on murine bladder cancer cell line (MBT-2).. Anticancer Res.

[6] Hammamieh R, Sumaida D, Zhang X, Das R, Jett M (2007). Control of the growth of human breast cancer cells in culture by manipulation of arachidonate metabolism.. BMC Cancer.

[7] Yoshimura R, Inoue K, Kawahito Y, Mitsuhashi M, Tsuchida K (2004). Expression of 12-lipoxygenase in human renal cell carcinoma and growth prevention by its inhibitor.. Int J Mol Med.

[8] Nie D, Hillman GG, Geddes T, Tang K, Pierson C (1998). Platelet-type 12-lipoxygenase in a human prostate carcinoma stimulates angiogenesis and tumor growth.. Cancer Res.

[9] Natarajan R, Esworthy R, Bai W, Gu JL, Wilczynski S (1997). Increased 12-lipoxygenase expression in breast cancer tissues and cells. Regulation by epidermal growth factor.. J Clin Endocrinol Metab.

[10] Kamitani H, Geller M, Eling T (1999). The possible involvement of 15-lipoxygenase/leukocyte type 12-lipoxygenase in colorectal carcinogenesis.. Adv Exp Med Biol.

[11] Laemmli UK (1970). Cleavage of structural proteins during the assembly of the head of bacteriophage T4.. Nature.

[12] Merrifield RB (1963). Solid Phase Peptide Synthesis. I. The Synthesis of a Tetrapeptide.. J Am Chem Soc.

[13] Otto A (1968). Excitation of surface plasma waves in silver by the method of frustrated total reflection.. Z Phys.

[14] Kretschmann E, Raether H (1968). Radiative decay of non-radiative surface plasmons excited by light.. Z Naturforsch.

[15] Nylander C, Leidberg B, Lind T (1982). Gas-detection by means of surface-plasmon resonance.. Sens Actuators.

[16] Lineweaver H, Burk D (1934). The Determination of Enzyme Dissociation Constants.. J Am Chem Soc.

[17] Wenzel SE, Kamada AK (1996). Zileuton: the first 5-lipoxygenase inhibitor for the treatment of asthma.. Ann Pharmacother.

[18] Skrzypczak-Jankun E, Zhou K, McCabe NP, Selman SH, Jankun J (2003). Structure of curcumin in complex with lipoxygenase and its significance in cancer.. Int J Mol Med.

[19] Wong BC, Wang WP, Cho CH, Fan XM, Lin MC (2001). 12-Lipoxygenase inhibition induced apoptosis in human gastric cancer cells.. Carcinogenesis.

[20] Skrzypczak-Jankun E, Zhou K, McCabe NP, Selman SH, Jankun J (2003). Structure of curcumin in complex with lipoxygenase and its significance in cancer.. Int J Mol Med.

[21] Whitman S, Gezginci M, Timmermann BN, Holman TR (2002). Structure-activity relationship studies of nordihydroguaiaretic acid inhibitors toward soybean, 12-human, and 15-human lipoxygenase.. J Med Chem.

[22] Somvanshi RK, Singh AK, Saxena M, Mishra B, Dey S (2008). Development of novel peptide inhibitor of Lipoxygenase based on biochemical and BIAcore evidences.. Biochim Biophys Acta.

[23] Jiang WG, Douglas-Jones A, Mansel RE (2003). Levels of expression of lipoxygenases and cyclooxygenase-2 in human breast cancer.. Prostaglandins Leukot Essent Fatty Acids.

[24] Liu XH, Connolly JM, Rose DP (1996). The 12-lipoxygenase gene-transfected MCF-7 human breast cancer cell line exhibits estrogen-independent, but estrogen and omega-6 fatty acid-stimulated proliferation in vitro, and enhanced growth in athymic nude mice.. Cancer Lett.

[25] Liu XH, Connolly JM, Rose DP (1996). Eicosanoids as mediators of linoleic acid-stimulated invasion and type IV collagenase production by a metastatic human breast cancer cell line.. Clin Exp Metastasis.

[26] Koutsilieris M, Reyes-Moreno C, Choki I, Sourla A, Doillon C (1999). Chemotherapy cytotoxicity of human MCF-7 and MDA-MB 231 breast cancer cells is altered by osteoblast-derived growth factors.. Mol Med.

[27] Singh AK, Parshad R, Pasi S, Madhavan T, Das SN (2011). Prognostic significance of cyclooxygenase-2 and response to chemotherapy in invasive ductal breast carcinoma patients by real time surface plasmon resonance analysis.. DNA Cell Biol.

[28] Singh AK, Kant S, Parshad R, Banerjee N, Dey S (2011). Evaluation of human LOX-12 as a serum marker for breast cancer.. Biochem Biophys Res Commun.

[29] Jiang WG, Douglas-Jones A, Mansel RE (2003). Levels of expression of lipoxygenases and cyclooxygenase-2 in human breast cancer.. Prostaglandins Leukot Essent Fatty Acids.

