# Supplementary material for: Structure Based Design and Synthesis of Peptide Inhibitor of Human LOX-12: In Vitro and In Vivo Analysis of a Novel Therapeutic Agent for Breast Cancer
Source: PLoS One. 2012 Feb 23;7(2):e32521. doi: 10.1371/journal.pone.0032521 (PMC3285689; doi:10.1371/journal.pone.0032521)
Supplement: Figure S1 — MTT assay of peptide AIRS (Negative control). (DOC) [file pone.0032521.s001.doc]

**Structure Based Design and Synthesis of Peptide Inhibitor of Human LOX-12:*In vitro* and *in* *vivo* Analysis of a Novel Therapeutic Agent for Breast Cancer**

**Abhay kumar Singh^1^, Ratnakar Singh^2^, Farhat Naz^3^, Shyam Singh Chauhan^2^, Amit Dinda^3^, Abhay Anand Shukla^2^, Kamaldeep Gill^1^, Vaishali Kapoor^4^, Sharmistha Dey^1^**

^1^ Department of Biophysics, ^2^ Department of Biochemistry, ^3^ Department of Pathology,

^4^ Department of Biotechnology, All India Institute of Medical Sciences, New Delhi, India

*Address for Correspondence:

Dr. Sharmistha Dey

Department of Biophysics

All India Institute of Medical Sciences

Ansari Nagar, New Delhi - 110029, INDIA

Tel: +91 - 11 - 26546435

Fax: +91 - 11 - 2658 8663

E - mail: [sharmistha_d@hotmail.com](mailto:sharmistha_d@hotmail.com)


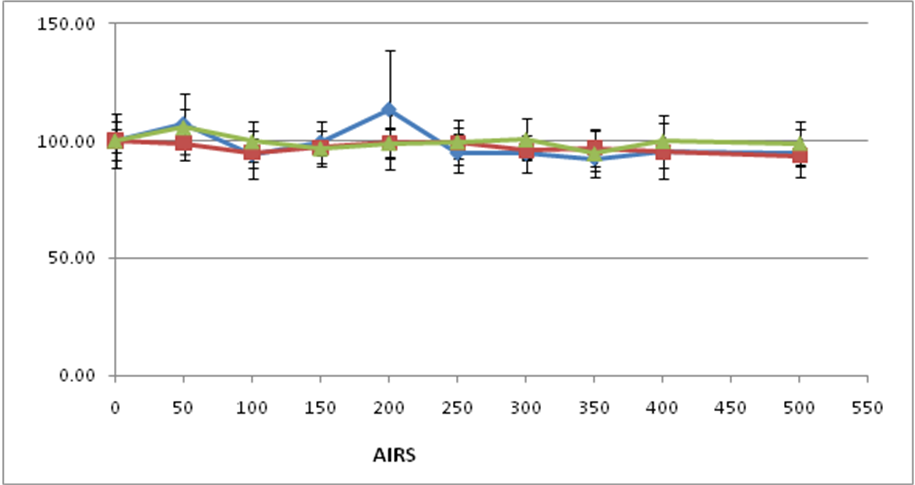


Supplementry figure (S 1):
